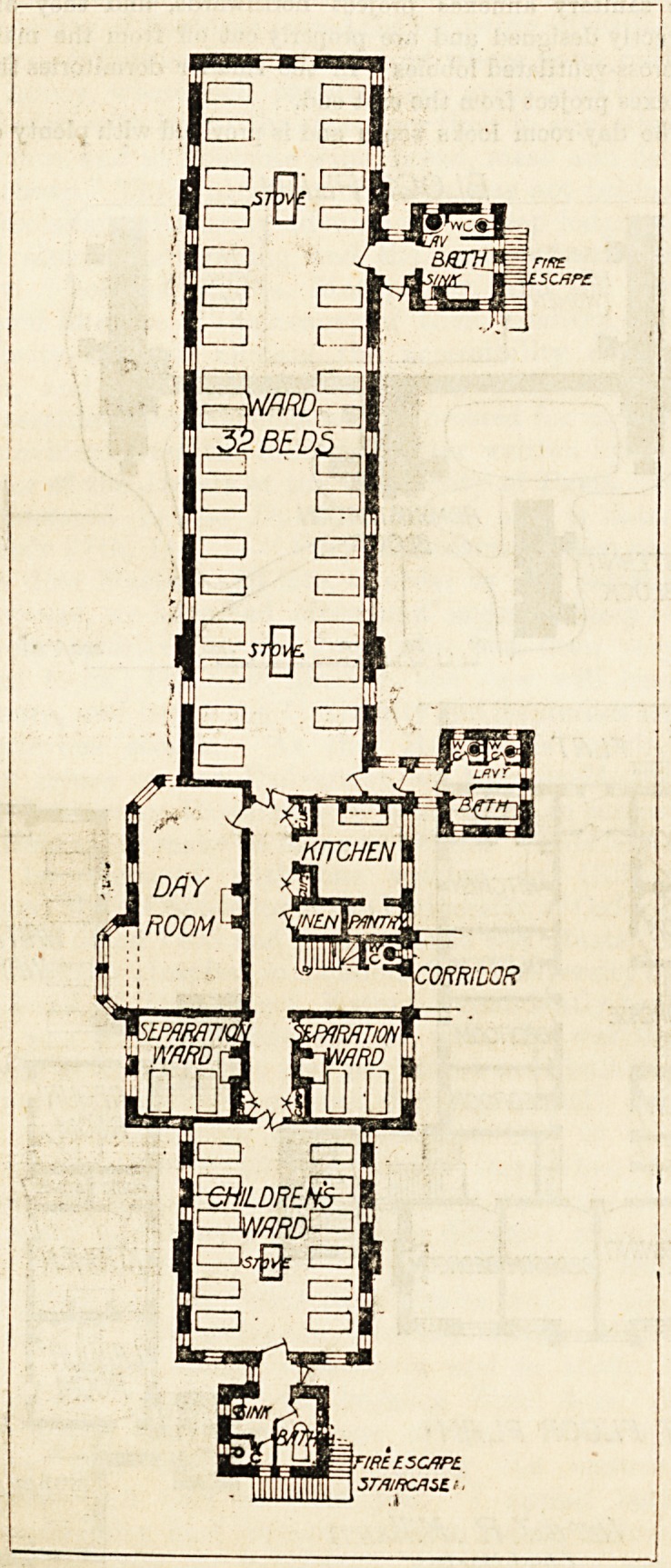# The Sunderland Union Infirmary

**Published:** 1903-10-03

**Authors:** 


### HOSPITAL ADMINISTRATION.

### CONSTRUCTION AND ECONOMICS.

### THE SUNDERLAND UNION INFIRMARY.

This infirmary consists of four blocks, three of which (the women's, the men's, and the administration) are connected by a corridor of about 100 yards in length. The pavilions run east and west, which is not the best aspect for a hospital ward, but it is probable that the site required this departure from the usual and better arrangement of north and south. These pavilions are two stories high and they contain 48 beds on each story. Each floor is subdivided into a large dormitory for 32 beds, and a smaller one for 12 beds, these wards being separated from each other by a day-room and a two-bedded isolation room on one hand, and by the ward kitchen, a staircase, and a second two-bedded room on the other hand. In the large wards and in relation to the windows the beds are placed in groups of two's—that is, each pair of beds has a window on each side of them, and although this arrangement may pass in a union infirmary, it could hardly be followed nowadays in a general hospital. The sanitary annexes project northwards, and they are correctly designed and are properly cut off from the main by cross-ventilated lobbies. In the smaller dormitories the annexes project from the east end.

The day-room looks south and is provided with plenty of light. The kitchen and the main staircase are conveniently placed, and each dormitory has a fire-escape staircase attached to its sanitary annexe. The worst features in these pavilions are the separation wards. They are not sufficiently removed from the rest of the block, and no cross-current of air can be obtained through them other than what may be got from fanlights placed over the doors, supposing they have fanlights, and even then the ventilation must be either from or towards the passage close by the door leading to the dormitory. The administration block is very well arranged, and it contains three floors and an attic floor.

The maternity block is entirely separate from the other pavilions; and it is throughout well-planned unless it be that the store-room space is deficient, a fault from which we fear experience will show that the other parts of the infirmary also suffer.

The buildings are warmed on the correct plan of open fireplaces, and hot-water radiators on the low-pressure system. The whole infirmary is of fireproof construction; and all the pipes for water, for heating, etc., are carried in subways to the various parts of the building. Electric light is used throughout.

The floors are of pitch pine, and this is a fairly good material if carefully selected, well-seasoned, and laid in narrow widths. The internal walls are finished in Parian cement and are painted and varnished. The angles are all rounded off.

Externally the walls are faced with Sherburn bricks.

The cost, exclusive of furnishing, was £35,000, or a little over £160 per bed.

The architects were Messrs. W. and T. R. Milburn, and the contractors were Messrs. Ranken, of Sunderland.

## Figures and Tables

**Figure f1:**
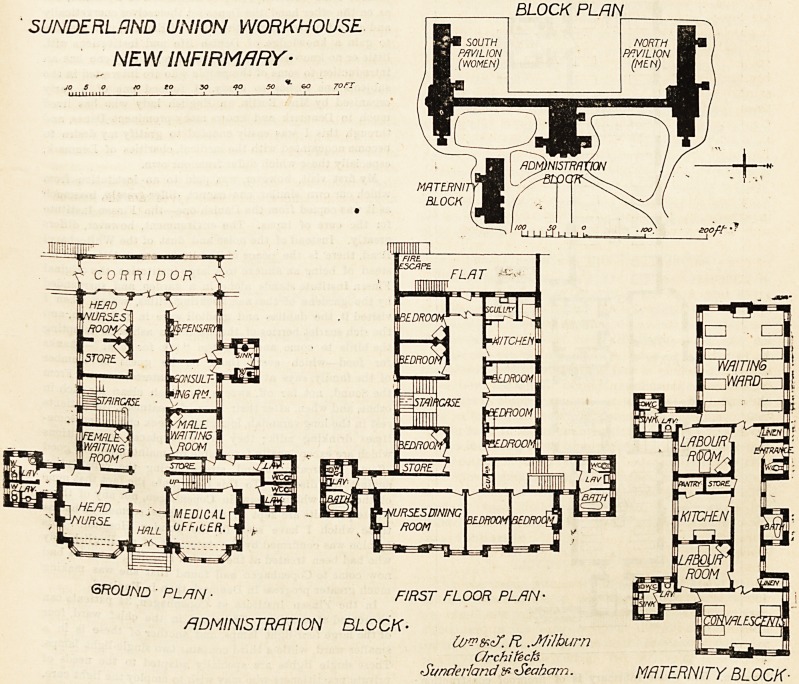


**Figure f2:**